# *Yersinia pestis*: New Evidence for an Old Infection

**DOI:** 10.1371/journal.pone.0049803

**Published:** 2012-11-28

**Authors:** Kirsten I. Bos, Philip Stevens, Kay Nieselt, Hendrik N. Poinar, Sharon N. DeWitte, Johannes Krause

**Affiliations:** 1 Institute for Archeological Sciences, University of Tübingen, Tübingen, Germany; 2 Laboratoire de Paléoanthropologie, École Pratique des Hautes Études, Université de Bordeaux, Bordeaux, France; 3 Center for Bioinformatics, University of Tübingen, Tübingen, Germany; 4 McMaster Ancient DNA Centre, Department of Anthropology, McMaster University, Hamilton, Ontario, Canada; 5 Michael DeGroote Institute for Infectious Disease Research, McMaster University, Hamilton, Ontario, Canada; 6 University of South Carolina, Departments of Anthropology and Biological Sciences, Columbia, South Carolina, United States of America; Natural History Museum of Denmark, Denmark

## Abstract

The successful reconstruction of an ancient bacterial genome from archaeological material presents an important methodological advancement for infectious disease research. The reliability of evolutionary histories inferred by the incorporation of ancient data, however, are highly contingent upon the level of genetic diversity represented in modern genomic sequences that are publicly accessible, and the paucity of available complete genomes restricts the level of phylogenetic resolution that can be obtained. Here we add to our original analysis of the *Yersinia pestis* strain implicated in the Black Death by consolidating our dataset for 18 modern genomes with single nucleotide polymorphism (SNP) data for an additional 289 strains at over 600 positions. The inclusion of this additional data reveals a cluster of *Y. pestis* strains that diverge at a time significantly in advance of the Black Death, with divergence dates roughly coincident with the Plague of Justinian (6^th^ to 8^th^ century AD). In addition, the analysis reveals further clues regarding potential radiation events that occurred immediately preceding the Black Death, and the legacy it may have left in modern *Y. pestis* populations. This work reiterates the need for more publicly available complete genomes, both modern and ancient, to achieve an accurate understanding of the history of this bacterium.

## Introduction

The recent demonstration that bacterial genome reconstructions are possible from archaeological material [1] presents a significant advancement in the field of infectious disease research by permitting an evaluation of the evolutionary history of human pathogens. Accurate quantification of genetic changes over time and rates of evolutionary change, however, are highly contingent upon our having an accurate representation of the diversity present in modern related organisms; hence, the evolutionary context of ancient pathogen genomes can only be properly interpreted if adequate sequence data are available and are reliably representative of extant pathogenic strains. Rapid and cost-effective DNA sequencing techniques have helped to increase the acquisition of such data, and while we eagerly await the addition of new pathogen genomes to publicly available databases, evolutionary histories can at best be extrapolated based on the limited data that are currently accessible.

Genomic comparisons of ancient and modern strains offer the best resolution for estimating rates of genetic change [2]. Such an approach was used in the analysis of our reconstructed ancient *Yersinia pestis* genome [1], where comparisons to all 18 complete modern genomes that are publicly accessible produced tighter rates of change than previous estimates, thus yielding more accurate divergence times on the phylogenetic tree [3], [1]. This method introduced an inherent limitation, however, since our exclusive use of available modern genomes likely encapsulated only a fraction of the true genetic diversity present amongst extant *Y. pestis* strains. To identify possible evolutionary events missed by our genomic analysis, we re-evaluated our reconstructed ancient *Y. pestis* sequence by comparing it against single nucleotide polymorphism (SNP) data publicly available for 289 *Y. pestis* strains at over 600 positions [3]. Although this method does not provide the resolution necessary for reassignment of our molecular clock values, it could provide a qualitative indication of phylogenetic signals that were lost via our original, more conservative analytical approach based strictly on complete genomes.

## Methods

We downloaded the raw SNP data of Morelli et al. [3] from the European Bioinformatics Institute’s ArrayExpress database (http://www.ebi.ac.uk/ArrayExpress) with accession number ID E-MTAB-213, consisting of 1217 SNPs in 289 strains. These were joined with the SNP data of Bos et al. [1] that characterise 1761 SNPs in 27 strains. The consolidated dataset resulted in a total of 946 SNPs in 311 strains.

All positions were assigned relative to the *Y. pestis* CO92 strain (NC_003143.1). This dataset carries several missing calls in individual strains or ambiguous calls that are likely attributed to sequencing issues. Based on these 946 SNP positions we then computed a Maximum Parsimony (MP) tree using MEGA 5 with 200 bootstrap replicates [4]. We chose the partial deletion method with a 95% cut off in MEGA 5 that removed 310 SNPs that had insufficient data before the MP-tree calculation. The resultant tree is, therefore, generated from the 636 remaining SNPs. Table S1 shows the sequence data for all 946 SNPs and identifies those that were either retained (Table S1a) or removed (Table S1b) by the 95% partial deletion filter. As in [1], we used the *Y. pseudotuberculosis* genome as our outgroup.

**Figure 1:**
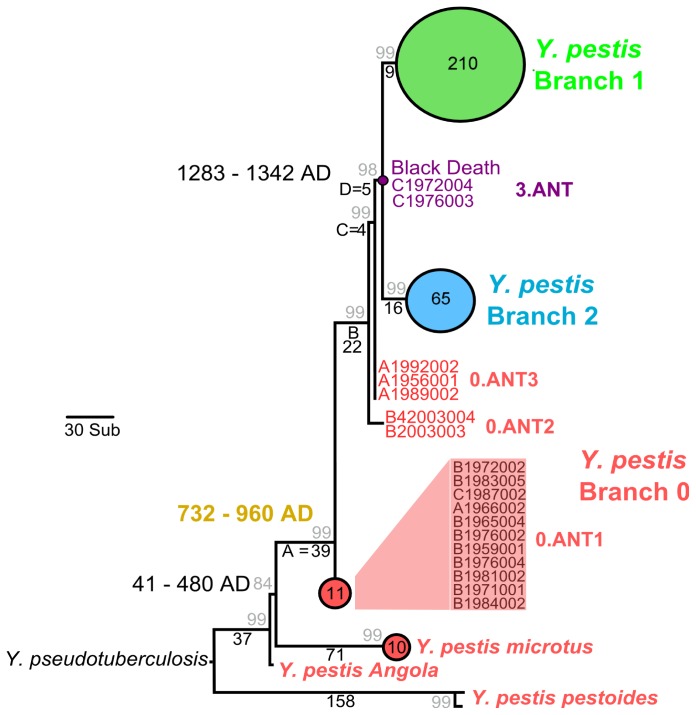
Maximum parsimony (MP) tree showing the relative phylogenetic placement of 311 *Y. pestis* strains. Branch 1 and Branch 2 designations as well as *Y. pestis* group names follow those defined in [9]. Dates in black denote those generated via full genomic analysis [1], whereas dates in gold are inferred here from relative branch lengths.

To infer the MP-tree we used the close-neighbour-interchange method. Inferred branch lengths were used to estimate a relative divergence time of the intermediate clade between the divergence of the rodent pathogen *Y.pestis microtus* (Figure 1, branch A) and the ancient Black Death cluster (Figure 1, branches B, C, and D). The ratio of branch length A to the total branch length A+B+C+D gives a divergence for the intermediate cluster of F = A/A+B+C+D = 0.5571 times the total distance. The approximate minimum and maximum divergence times of the intermediate *Y.pestis* cluster were calculated by multiplying our value for F by the distance between the two minimum and the two maximum ages of *Y.pestis microtus* (41 AD –480 AD) and the Black Death (1283 AD –1342 AD).

## Results and Discussion

Our MP tree (Fig. 1, Fig. S1) confirms the result shown previously that our ancient genome essentially falls at the root of all *Y. pestis* commonly-associated with human infection, namely the branch 1 and branch 2 strains [1]. Our previous full genome analysis revealed two SNPs common to the branch 1 family that distinguished our ancient sequence from the branch 1 and branch 2 root. Neither of these SNPs, however, are included in the current tree construction since one was absent in the Morelli et al. dataset [3] and was thus removed when the intersection of the two datasets was generated, and the other was removed by the 95% partial deletion filter in MEGA 5. Removal of these two positions makes the ancestral sequence sit directly at the root of the branch 1 and branch 2 strains.

Regardless, our analysis shows three novel observations that become apparent by our inclusion of SNP data from the additional *Y. pestis* strains. First, there is a cluster of 11 strains that diverge at a position predating the Black Death. These strains correspond to sequences in the 0.ANT1 *Y. pestis* group defined in Morelli et al. [3] (Fig. 1, Fig. S1). Our tree shows this group to consist of a single branch, whereas the network of Morelli et al. [3] shows it to be represented by three branches. Again, this discrepancy arises from our conservative approach of using the intersection of both the Morelli et al. and Bos et al. SNP datasets in combination with our partial deletion filter. High coverage full genomic data will be helpful to resolve the degree of genetic diversity present in these strains. It is not currently known if these sequences represent strains that are pathogenic to humans, though the placement of their divergence provisionally suggests a radiation event possibly resulting from a distinct epidemic occurring significantly in advance of the Black Death. Based on similarity in mortality levels, geographic distribution, and recorded symptoms, historians have long suspected that the Plague of Justinian (542–740 AD) might have been caused by the same infectious agent as that responsible for the 14^th^-century Black Death [5]. Since several publications have implicated *Y. pestis* as the principal cause of the Black Death by phylogenetic assignment and evaluation of DNA quality [1], [6], [7], the possibility that the Plague of Justinian may have been responsible for the deep cluster we observe here carries some legitimacy. This is further supported by the placement of the cluster approximately half the distance between the Black Death (1283–1342 AD) and the ancestral rodent strain *Y. pestis microtus*, which is suspected to have diverged from the soil-dwelling *Y. pseudotuberculosis* root approximately 2000 years ago (41–480 AD). Our cursory dating analysis based on relative branch lengths reveals a divergence time of 733–960 AD for this cluster, thus placing it in a phylogenetic position expected for a *Y. pestis* radiation event roughly coincident with the Plague of Justinian. We regard this as an important observation since a *Y. pestis* involvement in the plague of Justinian seemed unlikely from our previous whole genome analysis [1]. Confirmation of their potential for human infection via isolation of one of these strains, or an as yet uncharacterised close relative, from an infected patient would be helpful to understand their possible relationship to the plague of Justinian.

Second, there is a small cluster of *Y. pestis* sequences that diverge immediately predating the Black Death, all of which define the 0.ANT3 *Y. pestis* group [3] (Fig. 1, Fig. S1). Again, it is not known whether these in fact represent *Y. pestis* sequences that could be pathogenic to humans; however, the most parsimonious explanation holds that this cluster represents a genetic diversification event immediately preceding the disease’s arrival in Europe. Though this could have occurred either in human populations or in rodent populations, it most likely took place in East Asia. Genetic evidence is accumulating in support of the view long held by historians that the disease responsible for the Black Death likely emerged in China [3], [8], even though adequate historical evidence is currently lacking to disclose the precise location. This notion is supported by the fact that the strains in this cluster were all isolated from China [3]. Regardless, this diversification event likely gave rise to the strain(s) responsible for the high mortality in the European pandemic of 1347–1351 AD.

Third, the ancient strain obtained from medieval London clusters with two modern strains of the 3.ANT *Y. pestis* group [3] (Fig. 1, Fig. S1) based on SNP profiles, thus revealing that the variant responsible for the Black Death is identical to certain modern strains of *Y. pestis* for the 636 positions considered in this analysis. Full genomic data are, however, not currently available for these strains; thus, we cannot determine the full extent of their similarity with the ancient genome, and it is likely that the two modern strains discussed here possess additional derived positions to distinguish them from the ancient sequence. In addition, the genetic architecture of these modern strains is currently unknown, and since little is known is about the structure of the ancient genome, we cannot currently assess similarities in gene order between the strains.

We acknowledge that the above conclusions have yet to be confirmed via a more robust full genomic comparison of *Y. pestis* strains, both contemporary and historical. Specifically, *Y. pestis* data from human mass burials dating to the Justinian era may hold pertinent information to permit a more thorough evaluation of the evolutionary history of this notorious human pathogen.

## Supporting Information

Figure S1 **Maximum parsimony tree showing identification names for all** ***Y. pestis*** **sequences considered in this analysis.** Branch and group designations match those defined in [9]. Sample identification names match those in the ArrayExpress (E-MTAB-213) dataset. “E. Smith.” refers to the East Smithfield Black Death sequence described in [1].
(TIF)

Table S1 **Sequence data for all SNPs generated from the intersection of the Bos et al.** [**1**] **and Morelli et al.** [**3**] **datasets.** Table S1a shows those retained by the 95% partial deletion filter in MEGA 5, and Table S1b shows those removed by the filter.
(XLSX)
