# Supplementary figures and images for: Yersinia pestis: New Evidence for an Old Infection
Source: PLoS One. 2012 Nov 28;7(11):e49803. doi: 10.1371/journal.pone.0049803 (PMC3509097; doi:10.1371/journal.pone.0049803)

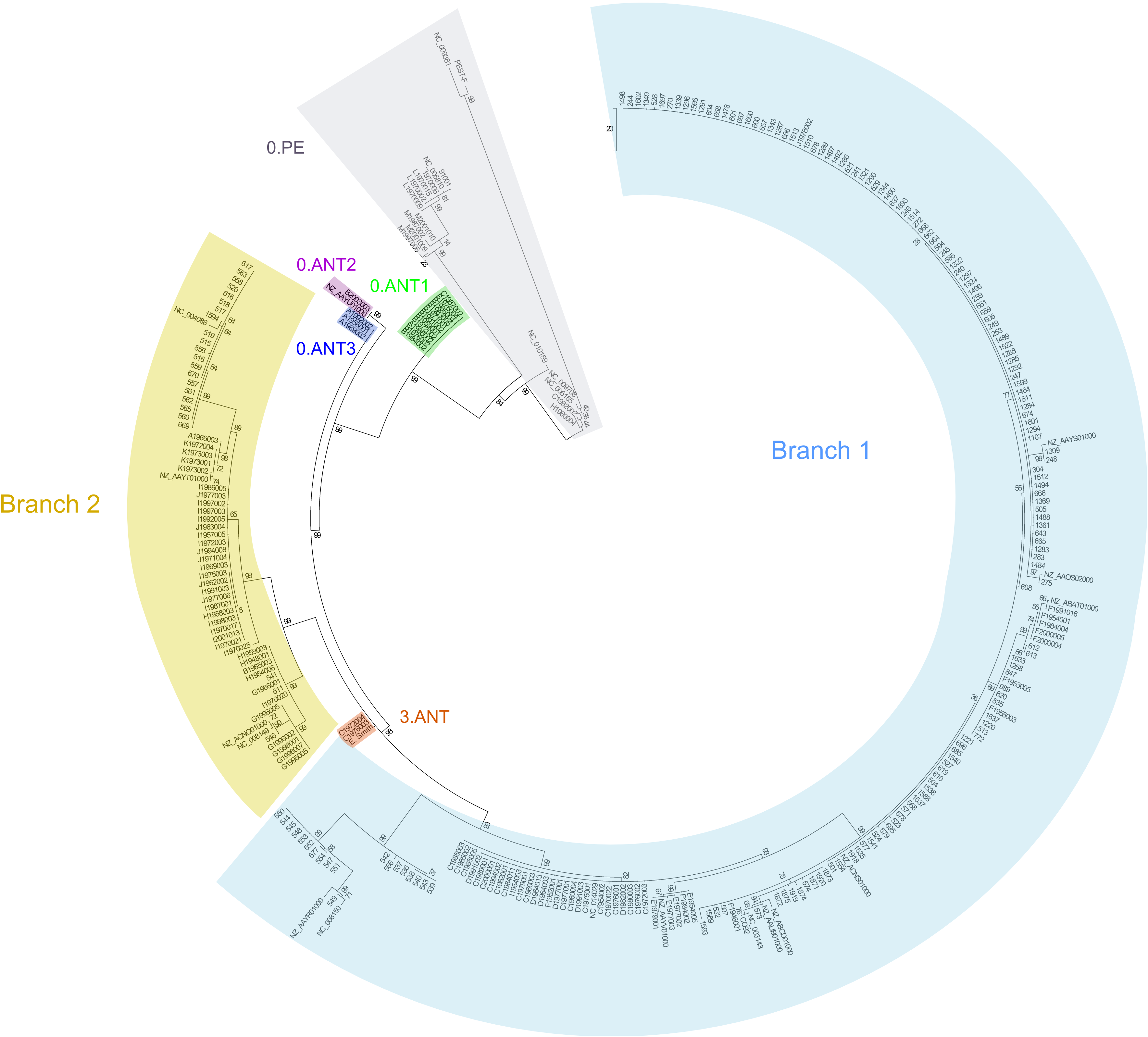

Supplement: Figure S1 — Maximum parsimony tree showing identification names for all Y. pestis sequences considered in this analysis. Branch and group designations match those defined in [9]. Sample identification names match those in the ArrayExpress (E-MTAB-213) dataset. “E. Smith.” refers to the East Smithfield Black Death sequence described in [1]. (TIF) [file pone.0049803.s001.tif]
